# Cutaneous and Skeletal Simultaneous Locations as a Rare Clinical Presentation of Tuberculosis

**DOI:** 10.1155/2015/618546

**Published:** 2015-04-30

**Authors:** Aida Pereira, Ana Miranda, Frederico E. Santo, Pedro Fernandes

**Affiliations:** ^1^Department of Infectious Diseases, Hospital Santa Maria, Lisbon, Portugal; ^2^Department of Internal Medicine, Service III-A, Hospital Pulido Valente, Lisbon, Portugal; ^3^Department of Orthopedics and Traumatology, Hospital Santa Maria, Lisbon, Portugal

## Abstract

Tuberculosis is a resurgent disease in most regions of the world, infecting one-third of the world's population and having a multisystemic involvement. Incidence of extra-pulmonary tuberculosis has increased in the last few decades as a result of the Human Immunodeficiency Virus (HIV) infection. The authors report a clinical case of the rare concomitant cutaneous and skeletal tuberculosis in an immunocompetent patient transferred from endemic area.

## 1. Introduction

Tuberculosis is one of the most ancient diseases across the world, caused by* Mycobacterium tuberculosis*, generally involving the lung where extra-pulmonary tuberculosis involvement occurs in 1/3 of the cases [1, 2].

The prevalence of the pulmonary and extra-pulmonary tuberculosis has increased in the last decades, attributed to immunosuppression related to HIV and other diseases like cancer, Diabetes Mellitus, end-stage chronic renal disease, congenital immunodeficiency's, and the high use of immunosuppressant drugs [3]. The resurgence has been attributed to a combination of HIV and other factors as increasing inner city deprivation, the arrival of immigrants from countries with a high incidence of tuberculosis, and dismantling of surveillance and contact-tracing services.

Musculoskeletal tuberculosis represents 1–3% of all cases, with the spine being most frequently affected, followed by the hip and knee [1, 2]. Cutaneous tuberculosis occurs in 1% of all tuberculosis cases and can mimic the clinical features of many other skin diseases [2, 4–6].

## 2. Case Presentation

Patient is a 25-year-old woman from Guinea-Bissau, West Africa, and was admitted with an 18-month history of dorsal back pain, asthenia, and significant weight loss followed in the last 8 months by loss of the ability to walk.

**Figure 1:**
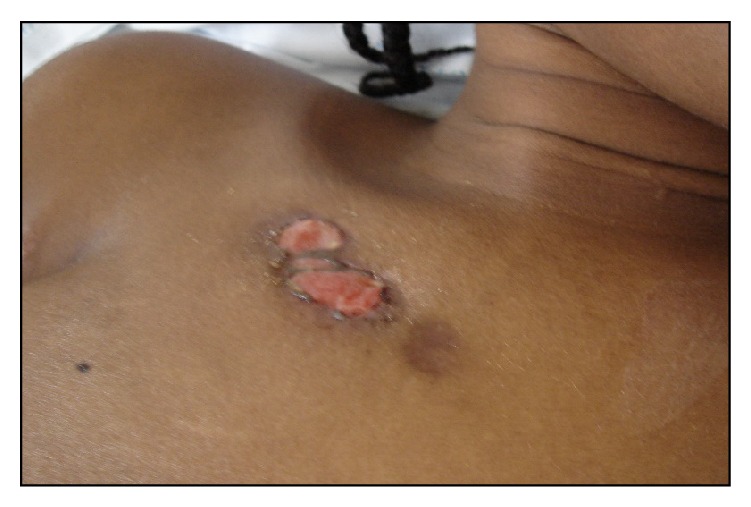
Clinical manifestation of cutaneous tuberculosis involving the right infraclavicular region and showing three lesions, one nodular and the others ulcerated.

**Figure 2:**
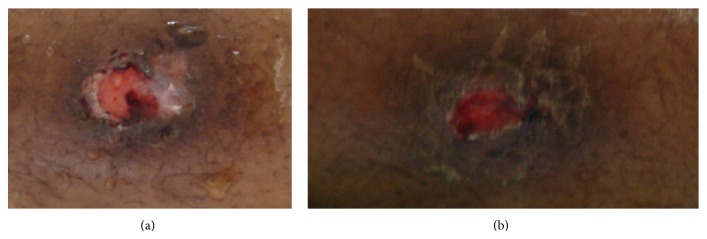
((a) and (b)) Clinical manifestation of cutaneous tuberculosis involving the left thigh and showing two lesions, one ulcerated (a) and the other in a healing phase (b).

**Figure 3:**
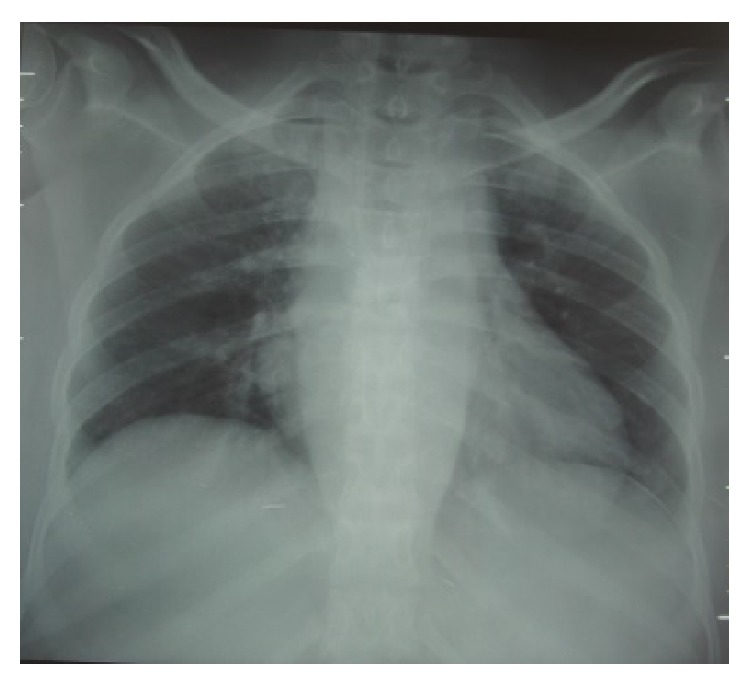
Chest X-ray showing enlargement of the mediastinum, consistent with tuberculous abscess mass.

**Figure 4:**
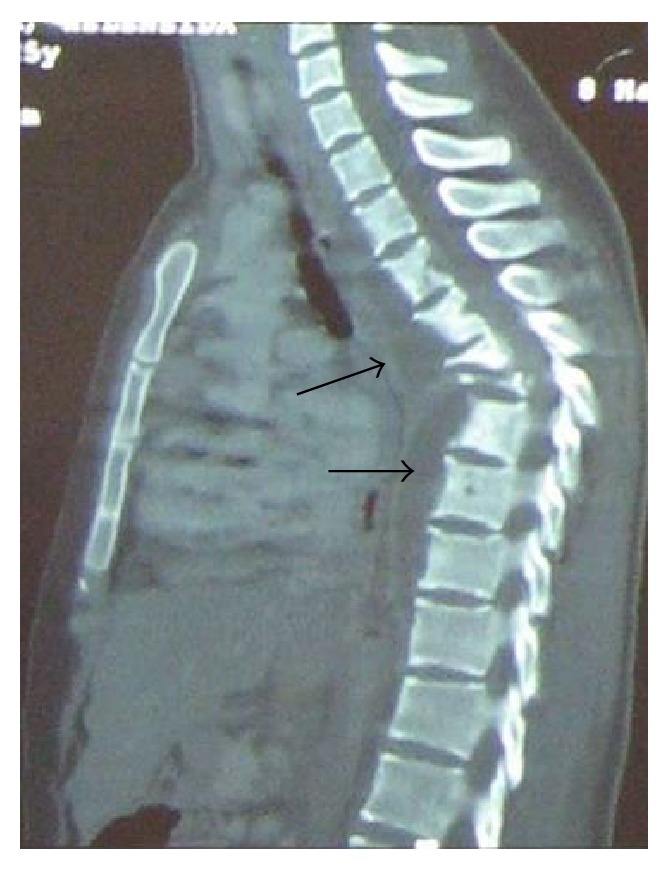
CT showing osteolytic lesions involving several vertebral bodies with severe kyphotic deformity, due to the collapse of the anterior spinal elements.

**Figure 5:**
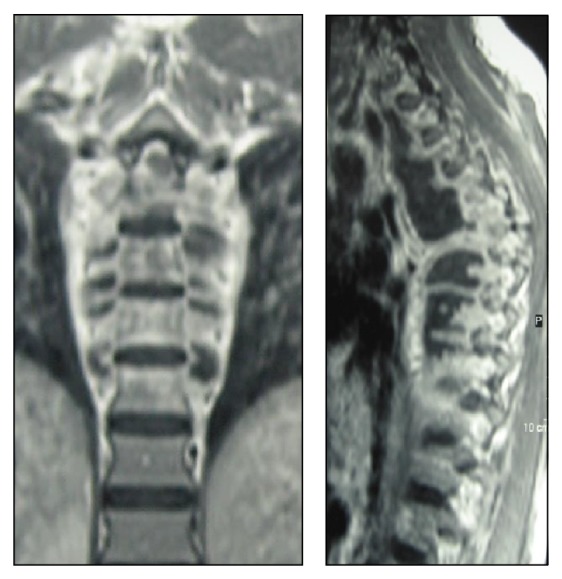
MRI with gadolinium showing a paravertebral abscess.

On her physical examination she presented with three skin lesions (two ulcerated and one nodular) involving the right subclavicle region and other two lesions on her left thigh (one ulcerated and the other in a healing phase) (Figures 1 and 2); her neurological exams exhibited an ASIA A paraplegia with a sensory level at D4. Patient was anaemic with hemoglobin of 11,2 g/dL with an erythrocyte sedimentation rate of 75 mm/h and a C-reactive protein of 5,26 mg/dL. Gamma globulin level was 44 g/dL and blood chemistry evaluation was normal. HIV and primary immunodeficiency testing were negative. A chest X-ray revealed an enlarged mediastinal shadow (Figure 3), and the spinal computed tomographic scan showed a large anterior and paravertebral abscess from D1 to D10 with significant compression and osteolytic lesions of vertebral bodies with a kyphotic deformity (Figure 4). Spinal magnetic resonance revealed significant compression of the spinal cord at the kyphotic apex and angulation of the column, anterior and paravertebral epidural abscess consistent with spondylodiscitis (Figure 5).

**Figure 6:**
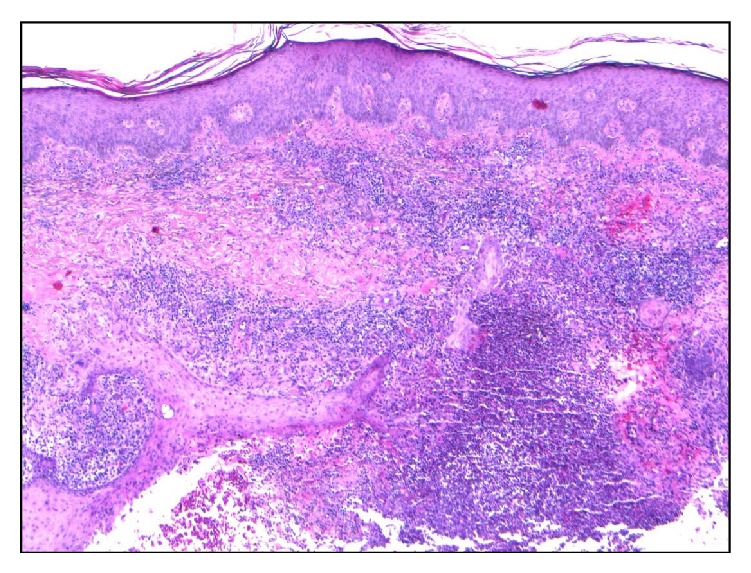
Histologic picture of the skin biopsy of one thigh lesion, showing pseudoepitheliomatous hyperplasia with suppurative granulomas.

Bone biopsy revealed granulomatous inflammation with few acid-fast bacilli. Skin biopsy of the lesions (Figure 6) revealed acanthosis and pseudoepitheliomatous, hyperplasia of the epidermis, and necrosis foci surrounded by a histiocytic and lymphocyte infiltrate with giant cells on the dermis, consistent with tuberculosis with negative Ziehl-Neelsen stain. The patient was started on quadruple antituberculosis drug regimen together with pyridoxine and steroids, with a progressive clinical improvement, including healing of the skin lesions. One month later, anterior surgical debridement and posterior arthrodesis of the spine were performed. Blood and urine cultures including cultures for mycobacteria all proved negative. Cultures of the material collected through bone biopsy revealed* M. tuberculosis*. The patient fulfilled 15 months on antituberculosis medication and a favorable outcome was reported with complete recovery of neurological deficits.

## 3. Discussion

Extra-pulmonary tuberculosis occurs in 10% of all cases of tuberculosis [7]. Cutaneous tuberculosis is frequently caused by* M. tuberculosis*, by* Mycobacterium bovis,* and rarely by the Calmette-Guerin bacillus [8]. Cutaneous tuberculosis is a relatively rare manifestation of tuberculosis, corresponding to 1-2% of all cases [9, 10]. Skin involvement may result from exogenous inoculation, spread from an adjacent focus, or a hematogenous spread from a distant focus as a part of the generalized hematogenous dissemination [11].

In the last two decades there was a decline in cutaneous tuberculosis incidence but, recently, it has appeared again associated with multidrug-resistant tuberculosis [4, 12] and the high incidence of HIV infection. The clinical picture of cutaneous mycobacterial infections, including tuberculosis, is highly variable, such as nodules, papulovesicular lesions, pustules, and ulcers [13]. Any unexplained skin lesions, especially, if nodular or ulcerative, may be due to tuberculosis.

Skeletal tuberculosis occurs in 10–15% of extra-pulmonary cases [1] as a result of past hematogenous foci, contiguous diseases, or lymphatic spread. The lower thoracic spine is involved most frequently, followed by lumbar spine [1, 14], and paraspinal cold abscesses develop in 50% of the cases. Epidural or psoas abscess can also complicate tuberculous spondylitis. The clinical presentation of pulmonary tuberculosis with bone or skin involvement has been reported in 50 to 65% of the cases; however simultaneous presentation of bone and skin is not a common feature of tuberculosis [1]. Kumar et al., described bone disease in four patients out of 75 cases of cutaneous tuberculosis [5].

Diagnosis of cutaneous tuberculosis can be confirmed by coloration method on biopsy, although results are often negative since skin lesions have often low counts of bacillus [1, 14]. Host immunological state and way of infection may define the clinical presentation of cutaneous tuberculosis and the results of diagnostic exams [7]. In immunocompetent individuals with the disease acquired by direct inoculation, the tuberculin test (Mantoux) is usually positive [15], being present in 90% of bone tuberculosis cases [1].

Skin disease acquired by endogenous transmissions is usually associated with variable degrees of immunosuppression especially with cutaneous abscesses and miliary tuberculosis. As such, response of tuberculin test can be negative as a result of low cell-mediated immunity and a decrease of the late hypersensitivity reactions [15]. The polymerase chain reaction of* M. tuberculosis* increases the probability of diagnosis in suspected cases [14].

In bone tuberculosis, the diagnosis is confirmed by histological confirmation on bone biopsy or pus from abscesses. The histological findings are very typical and cultures are usually positive. However, as in cutaneous tuberculosis, the lesions have low counts of bacillus, which hinders the demonstration or the growth of mycobacteria [1]. In our patient, diagnosis was obtained from visualization of acid-fast bacilli on the direct exam of pus from a paravertebral abscess, posteriorly confirmed on culture mediums.

Cutaneous and skeletal tuberculosis should be included in the diferential diagnosis of other skin and muscle-skeletal disorders, especially in populations of endemic regions of tuberculosis [15].
